# Giant Cell Arteritis: Updates and Controversies

**DOI:** 10.3389/fopht.2022.848861

**Published:** 2022-03-17

**Authors:** Erin Yu, Jessica R. Chang

**Affiliations:** University of Southern California Roski Eye Institute, Keck School of Medicine of USC, Los Angeles, CA, United States

**Keywords:** giant cell arteritis (GCA), MRI black blood, arteritic anterior ischemic optic neuropathy (AAION), epidemiology, vasculitis, temporal artery biopsy

## Abstract

**Abstract:**

Giant cell arteritis (GCA) is a systemic granulomatous vasculitis affecting the medium and large-size arteries, and may present with a range of ophthalmic findings. This review will cover GCA epidemiology, pathophysiology, clinical presentation, diagnostic workup, and treatment.

**Epidemiology and Pathophysiology:**

GCA is commonly found in elderly patients and individuals of Scandinavian descent. Recent publications suggest it may be more common in African Americans and Hispanics than previously thought. It is very rare in Asian and Middle-Eastern populations, and there is little data regarding African populations. Genetic studies have identified increased risk associated with HLA-DRB1*04. Rather than a response to a specific antigen such as varicella zoster virus, current immunology research suggests that GCA results from changes associated with the aging immune system.

**Clinical presentation to Ophthalmology:**

Arteritic anterior ischemic optic neuropathy is the most common ophthalmic manifestation of GCA, but central or branch retinal artery occlusion, ophthalmic artery occlusion, cranial neuropathies causing diplopia, and more rarely anterior segment ischemia and anisocoria may also occur. Clinical testing including visual field testing, OCT, OCT-A, ICG and fluorescein angiography can be helpful in suggesting a diagnosis in addition to the clinical exam.

**Diagnostic Workup:**

GCA is ultimately a clinical diagnosis, but it is usually supported with lab results, pathology, and/or imaging. Temporal artery biopsy (TAB) remains the gold standard diagnostic test although its sensitivity is debated and practice patterns still vary with respect to sample length and whether unilateral or simultaneous bilateral biopsies are performed. Some studies have reported higher sensitivity of ultrasounds over TAB, with added benefits of time efficiency and cost effectiveness, promoting the diagnostic use of ultrasounds. MRI and even PET CT protocols offer additional options for less invasive diagnostic testing.

**Treatment:**

Vision-threatening GCA is treated acutely with emergent admission for intravenous methylprednisolone, and long-term high dose oral corticosteroids remain the standard of care, despite common and sometimes serious side effects. The use of steroid-sparing alternatives such as tocilizumab is becoming more common and additional agents are being investigated.

## Introduction

Giant cell arteritis (GCA) is a systemic granulomatous vasculitis that affects medium and large arteries in patients over the age of 50 (1). GCA commonly presents with non-specific constitutional symptoms including fever, night sweats, anorexia, weight loss, fatigue, or myalgia. About half of patients diagnosed with GCA have polymyalgia rheumatica (PMR), a relatively common rheumatic disease of the elderly that presents with cervical, shoulder, and hip pain and stiffness (2). PMR and GCA share similar epidemiology and are both typically managed with glucocorticoids. GCA is classically distinguished by cranial ischemia-related symptoms such as headache, jaw claudication, and scalp tenderness, although large-vessel involvement leading to complications such as aortic aneurysm is becoming more widely recognized (3). This “extracranial” or “large-vessel” form of GCA can be more challenging to diagnose and to distinguish from PMR. Advances in diagnostic imaging as well as immunology are shedding new light on this form of GCA, and can also be helpful in the classic (“cranial”) form of GCA. The classic cranial presentation of GCA is frequently associated with ophthalmic manifestations.

GCA poses a significant disease burden due to its potential risk of permanent visual loss, as well as frequent adverse side effects from long-term glucocorticoid treatment. One systematic literature review predicted that by 2050, more than 3 million people will be diagnosed with GCA due to the aging population, leading to visual impairment in 500,000 individuals in Europe, North America, and Oceania (4). In the United States alone, the projected cost from visual impairment due to GCA will exceed US$76 billion by 2050 (4). Prompt diagnosis and management of this disease may help reduce the burden of visual impairment. New research has challenged our understanding of the epidemiology, diagnosis, and treatment of GCA, and will hopefully promote better recognition, management and outcomes for patients.

## Epidemiology and Pathophysiology

Most epidemiological studies support relatively higher incidence rates of GCA in white populations, especially those of Scandinavian descent, with increased risk in women and individuals over 50 years of age (5). Incidence rates of GCA have been shown to vary amongst different European countries, with generally higher incidence rates in Northern European countries (**Table 1**). It has traditionally been understood that GCA is rare in non-white patient populations. However, most epidemiological studies of GCA have generally consisted of predominantly white populations in Europe and North America, and data regarding GCA incidence in other races and ethnicities remain limited. One study from Japan estimated a prevalence of 1.47 per 100,000 over age 50 (18) (for comparison, prevalence in Minnesota is 204 per 100,000 over 50 whereas incidence is 19.8 per 100,000 over 50), but other studies from Asia, Africa, and the Middle East are generally limited to case reports or retrospective reviews from single institutions (19, 20). For example, a study from the UK found 1 of 26 positive biopsies over 8 years was from a South Asian patient (21); another study from Mumbai found 21 patients over 15 years (22). Studies from a large center in Saudi Arabia imply a low incidence of GCA in the Arab population based on low rate of positive TAB (7/102 biopsies over 22 years) (23), and a low rate of arteritic anterior ischemic optic neuropathy (AAION) compared to nonarteritic anterior ischemic optic neuropathy (NAION) (24).

**Table 1:** Incidence of GCA in various populations.

Country	Reported Race/Ethnicity	Criteria for GCA diagnosis	Incidence per 100,000 (over the age of 50)	Reference
Denmark	*	Clinical and pathologic diagnosis	20.4	Elling et al. (6)
France	*	Typical signs and symptoms	9.4	Barrier et al. (7)
Iceland	*	1990 ACR Classification Criteria, TAB, and clinical diagnosis	27	Baldursson et al. (8)
Italy	*	Positive TAB	5.8	Catanoso et al. (9)
New Zealand	*	Positive TAB	12.7	Abdul-Rachman et al. (10)
Norway	*	1990 ACR Classification Criteria, TAB, and imaging	16.8	Anderson et al. (11)
Spain	*	Clinical and pathologic diagnosis	10.13	Gonzalez-Gay et al. (12)
Spain	*	1990 ACR Classification, TAB, clinical diagnosis	2.2	Romero-Gómez et al. (13)
United Kingdom	*	Typical signs and symptoms	2.2	Smeeth et al. (14)
USA	African American	Clinical and pathologic diagnosis	0.36	Smith et al. (15)
USA	African American	Positive TAB	3.1	Gruener et al. (16)
USA	Olmsted County (population primarily of Northern European descent)	1990 ACR Classification, TAB, clinical diagnosis, and imaging	19.8	Chandran et al. (17)

*No specific racial/ethnic categories were reported.

GCA, Giant cell arteritis; TAB, Temporal artery biopsy; ACR, American College of Rheumatology.

Given the ethnic and racial diversity in North America, estimates based on racial and ethnic groups within the United States have been used to compare populations: for example, a retrospective study of GCA from San Francisco reported the incidence of GCA in Asians was about 20 times less than in white patients (25). Interestingly, the evidence for GCA in the African American population has shifted over the years. An oft-cited study assessing the incidence of GCA in Shelby County, Tennessee from 1971-1980 reported the incidence of GCA to be 7 times greater in white patients compared to black patients (15). In contrast, another retrospective study conducted from the southern United States from 1974-1984 reported 13 out of 27 cases of GCA to be in black women (26). A larger study comparing the incidence of GCA in black and white patients in Baltimore from 2007-2017 supports the idea of GCA being more common than previously thought in black populations (or at least African Americans), with an estimated incidence of 3.1 per 100,000 over age 50 for black patients and 3.6 per 100,000 over age 50 for white patients (16). A recent multicenter study evaluated differences in presenting symptoms of GCA between 32 African American patients and 84 Caucasian patients with biopsy-proven GCA, and found that African American patients had significantly higher rates of headache, neck pain, anemia, and eye pain, and lower rates of jaw claudication and acute vision loss (27). To what extent these incidence estimates and clinical features can be extrapolated to African black populations is unknown, as the African American genome contains variable amounts of European (and other) genetic markers.

The incidence of GCA in Hispanic patients also remains a point of controversy. The first study in the US to evaluate GCA in Hispanic patients was a small retrospective study from Los Angeles which found that none of the 40 self-identified “Latino” Hispanic patients had positive temporal artery biopsy (TAB) results, compared to 19 of the 66 white patients—in this study patients were categorized as Hispanic or white, but not both (28). More recently, a study from Miami reviewed TAB results from 1996-2002 (29), distinguishing race and Hispanic ethnicity as two separate concepts, and reported similar prevalence and clinical course of GCA among Hispanic and non-Hispanic patients. It must be noted, however, that all of the patients with positive TAB identified their race as white. This difference in definition highlights the confounding problem that “Hispanic” reflects a diverse range of different racial groups and genetic heritages, just as race labels such as black, white, and Asian reflect a wide diversity of backgrounds, and individual patients may belong to multiple categories. If white generally consists of those with European ancestry, it still does not capture the ten-fold differences in GCA incidence between different European populations, which have been well characterized.

Genetic and environmental influences have also been studied. The most consistent genetic association has been with HLA-DRB1*04 (30), but several other genes associated with cytokines, the innate immune system, and endothelial cells have been suggested to also have some influence, and there are also reports of familial GCA although this is rare (30, 31). Among environmental factors, seasonality and exposure to specific pathogens have been explored. Some studies suggest a seasonal variation with higher incidences of GCA in the summer months in Eastern Denmark (32), California (33), Southern Australia (34), and the UK (14). Other studies have reported higher incidence of GCA during the autumn and winter (35). Meanwhile, some other studies reported no significant correlation between GCA incidence and seasons (36, 37). Discrepancies in the results of these seasonal studies may be attributed to differences in the way the studies defined GCA (e.g. biopsy-proven or clinical diagnosis) or the definition of GCA disease onset (32).

Exposure to certain antigens has also been hypothesized to trigger GCA. In 2016 Gilden and Nagel proposed that varicella zoster virus (VZV) was a likely trigger, and they reported viral particles were found in TAB specimens, however most subsequent studies have found no relation between VZV exposure or VZV vaccination and GCA and many have failed to replicate the findings of VZV in TAB specimens, suggesting that the original finding may have been an artifact (38–40). Various other specific pathogens have been investigated, but it is more likely that at the population level there is some association with a wide range of preceding infections more generally, and not any one specific infectious agent (41).

Current understanding of the immunopathology of GCA implicates age-related dysfunction of the immune system and its interactions with the aging blood vessel wall rather than an immune response to any one specific antigen (31). When there is a constellation of genetic risk factors and environmental triggers, dendritic cells in the adventitia recruit T cells which leads to cytokine production, interferon gamma production, and inflammation including macrophages which can differentiate into giant cells (42). This inflammation remodels the vessel wall, leading to ischemia of the downstream organs. Proteins released by macrophages are being investigated as potential diagnostic and/or prognostic biomarkers that may be more specific than the erythrocyte sedimentation rate (ESR) and C-reactive protein (CRP) levels (43).

## Clinical Presentation to Ophthalmology

The general clinical presentation of GCA is infamously variable; classic temporal arteritis—the “cranial” phenotype–is the one most associated with ophthalmic symptoms, but up to 20% of patients may present to the ophthalmologist with “occult” GCA—vision loss but no classic systemic symptoms (44). The rate of ocular involvement ranges from 10-70% in various studies (44, 45). GCA is well known in ophthalmology for its risk of irreversible blindness due to arteritic anterior ischemic optic neuropathy (AAION), which has been reported in over 80% of patients who present with ocular involvement (3, 46). Transient visual symptoms such as amaurosis fugax may often precede permanent visual loss (44). As shown in Mahr et al, ophthalmologists were the second most common referring specialty (after primary care), highlighting the importance of ophthalmic symptoms in raising the suspicion for GCA (47).

**Figure 1:**
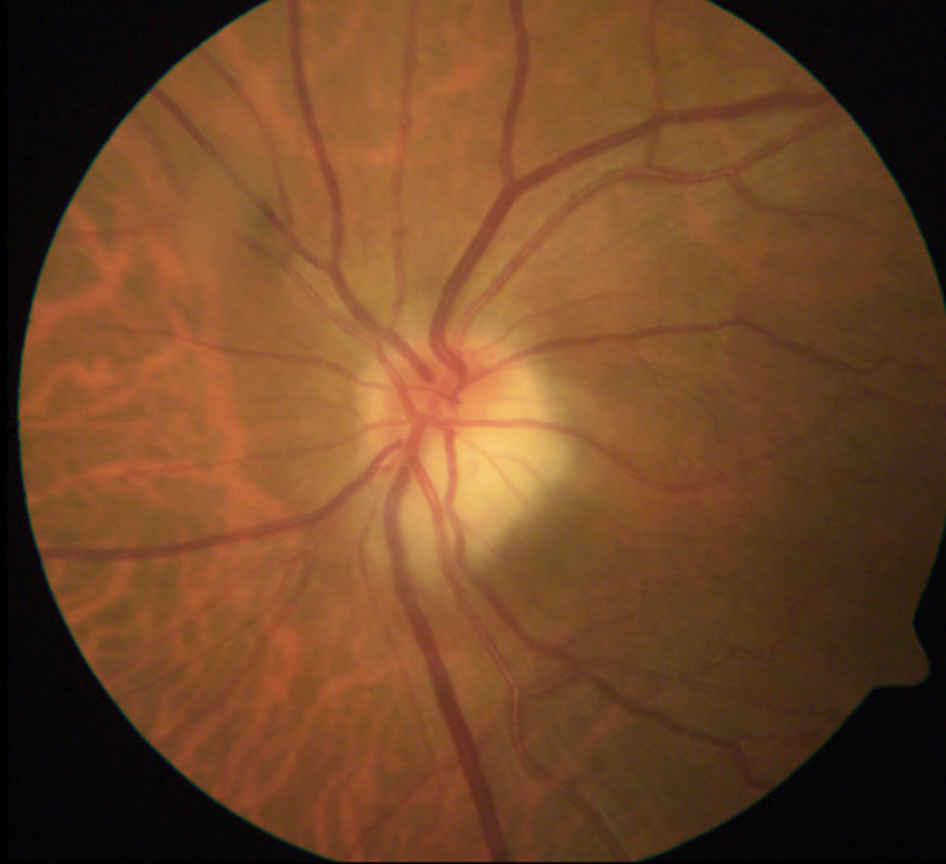
Acute AAION. Image courtesy of Kimberly Gokoffski, MD PhD.

**Figure 2:**
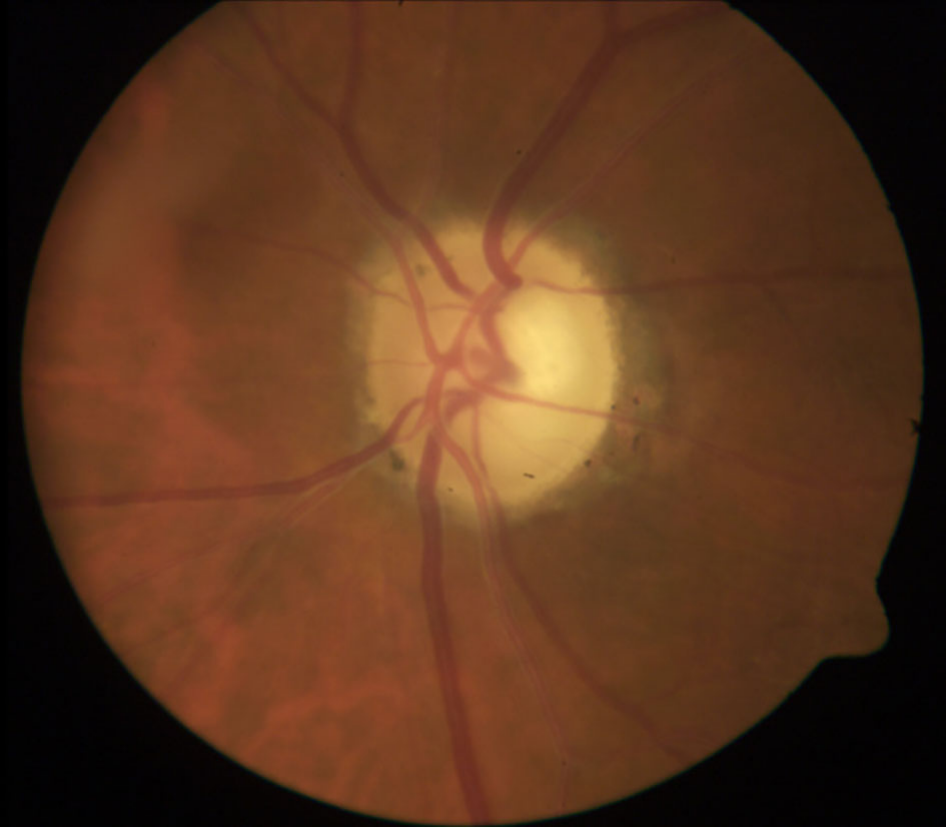
Optic atrophy with apparent cupping after resolution of acute AAION. The optic disc atrophy following AAION (but not NAION) may resemble severe glaucomatous cupping. The remaining rim is usually pale after AAION but pink in glaucoma. OCT has been studied as a way to differentiate between glaucoma and atrophy after AAION: one study showed that non-glaucomatous cupping exhibited lower mean retinal nerve fiber layer (RNFL) thickness in the nasal quadrant, lower average macular thickness, and lower macular volume (49). Image courtesy of Kimberly Gokoffski, MD PhD.

AAION is characterized by ischemia of the posterior ciliary arteries (PCA) and loss of blood supply to the optic nerve head, with the medial branch of the PCA often affected (46). In the acute phase, AAION classically presents with severe vision loss (no light perception in up to 19% (48), and a swollen and chalky-white optic disc (**Figure 1**], progressing to optic atrophy within 6 to 8 weeks (**Figure 2**] (50). Patients with GCA may also present with arteritic posterior ischemic optic neuropathy (PION), which is less common and not visible on ophthalmoscopy (50). Up to 16% of patients with vision loss from GCA may have ischemic stroke (51), most often involving the vertebrobasilar territory (52). More rarely, cerebral ischemic lesions may result in visual loss (19).

GCA may involve occlusion of the central retinal artery (CRAO) in up to 14% of patients, or it may occlude the cilioretinal artery or other branches (46). Cotton wool spots may be seen in up to 30% of GCA patients who suffer early vision loss (46). Anterior segment ischemia and anisocoria are rare manifestations of GCA and may present with ocular hypotony, corneal edema, iris ischemia, and pupillary abnormalities (44). Ischemia of cranial nerves III, IV, and VI may result in diplopia, which can occur in up to 6% of patients, and more rarely ischemia of cranial nerve VII can present with eyelid position changes (51).

Visual field testing may be helpful in demonstrating the severity of visual field loss in ischemic optic neuropathy if the visual acuity is sufficient (e.g. 20/200 or better); typical AAION generally causes severe loss of visual field but a wide variety of patterns may be seen (51, 53). Visual field testing is helpful in identifying scotomas that the patient may not have noticed, for example, in the better seeing eye which may still have early ischemia. Fundus fluorescein angiography (FFA) or indocyanine green angiography (ICG) may exhibit significantly delayed filling of the choroid in patients with AAION (54). One study using FFA reported an average choroidal filling time of 69 seconds for patients with AAION, compared to an average of 5.8 seconds in normal subjects, or 5.5 seconds in nonarteritic AION (55).

Optical coherence tomography angiography (OCT-A), a non-invasive alternative to FFA and ICG, has also been shown to identify ischemic areas on the optic nerve head and reduction in vessel densities in patients with AAION (56). OCT-A may be more sensitive than FFA in its ability to visualize macular and radial peripapillary capillaries, but is not able to distinguish between AAION and non-arteritic anterior ischemic optic neuropathy (NAION) as well as FFA (56). A small case series suggested that changes in superficial microvasculature on OCT-A were not visible on FFA, although areas of choroidal hypoperfusion noted on FFA were not visible on deep laminar OCT-A segmentation (57). New advances on the horizon include laser speckle flowgraphy, which may improve our ability to distinguish acute AAION from NAION (58).

## Diagnostic Workup

The diagnosis of GCA is a clinical one, supported by lab results, pathology, and imaging. The ACR classification criteria for GCA have often been used for diagnosis although they were intended for research studies (59). Up to a quarter of GCA patients may have normal ESR, and C-reactive protein (CRP) may be a better inflammatory marker for clinical monitoring (3). Temporal artery biopsy (TAB) has traditionally been used for diagnosis but its sensitivity varies widely, and practice patterns vary over how and when to perform the biopsy and how to interpret the histopathology. Increasingly, ultrasound, MRI, and even PET CT are being used to evaluate for GCA (58).

Temporal artery biopsy (TAB) has conventionally been the gold standard for diagnosis, yet there are limitations pertaining to its low sensitivity and false-negative results. It is overall a very safe procedure, but potential risks include scalp necrosis, hematoma, infection, facial nerve damage, and rarely, cerebral ischemia (60). Clinical exam of the arteries is normal in about a third of positive TAB’s, and the presence of localizing symptoms on one side does not necessarily mean the biopsy will be positive on that side (3, 61). The range of sensitivity for TAB has varied widely across studies, from 24-94%, possibly depending on factors such as length and processing of specimens as well as timing of biopsy after initiation of steroid therapy (3, 62).

To improve the sensitivity, some studies have advocated for longer arterial specimens, and/or simultaneous bilateral specimens, as well as protocols for pathologic sectioning to more adequately sample the entire length of artery and reduce the chances of missing skip lesions (63, 64).

More recent studies have argued that shorter specimen lengths are sufficient, with post-fixation length as low as 0.5cm although most authors recommend at least 1cm (which may correspond to almost 2cm pre-fixation) (62, 65). Studies of discordance have suggested rates of 3-9%, and surgeons continue to vary in whether they perform initial bilateral or unilateral biopsy (61). The American College of Rheumatology (ACR) recommends an initial unilateral temporal artery biopsy (TAB) of >1 cm within 2 weeks of starting oral (58). Current recommendations from both the British Society for Rheumatology (BSR) and European League Against Rheumatism (EULAR) suggest >1 cm biopsies corresponding to a post-fixation length of at least 0.7 cm, and do not routinely recommend taking a biopsy of the contralateral artery (66, 67).

Most studies agree, at least, that the specificity of TAB is quite high, although there are controversies regarding pathologic findings, particularly related to the concept of “healed arteritis” which can be difficult to distinguish from other age-related vascular changes (68). Classic findings of GCA on biopsy include, but are not limited to, histiocytes, epithelioid, and multinucleated giant cells between the intima and media, transmural inflammation marked by lymphocytic infiltrates, fragmentation of the internal elastic lamina, and intimal hyperplasia (62). However, multinucleated giant cells are only seen in about half of all positive specimens (3). Some studies have also used CD68, a marker for transmembrane glycoproteins on monocytes and tissue macrophages, in determining if a TAB is positive in the absence of multinucleated giant cells (69). Pathologists may also have discordant opinions on assessing histological features, as one study reported an intraclass correlation coefficient of 0.62 for TAB diagnosis (95% CI 0.49-0.76) (40). “Healed” arteritis is another controversial topic that is still not completely understood, where the biopsy is not normal but does not have active disease, and these may reflect a heterogeneous group of patients (68, 70). Furthermore, some have questioned whether TAB results actually change management—one studied reported that 87% of TAB negative patients remained on therapy due to clinical judgement (71).

Perhaps the most important factor for improving sensitivity in TAB (and other diagnostic testing) is pretest probability. Risk stratifying algorithms have been developed (72, 73), some are used in fast-track clinics to improve diagnostic accuracy and the sensitivity of screening ultrasound (73, 74). Because of the high incidence of false negative TAB, it has low negative predictive value, and various imaging modalities have been increasingly used to support (or exclude) the diagnosis of GCA. Imaging techniques such as temporal artery ultrasound (TAUS), which also include axillary arteries and color doppler imaging, have become first-line diagnostic tests instead of TAB in the 2018 European League Against Rheumatism (EULAR) recommendations (67). Positive ultrasounds in GCA present with a “halo sign” of dark hypoechoic area around the area of mural edema in the vascular wall (75).

A meta-analysis of 8 studies (605 patients) showed TAUS had sensitivity of 77% and specificity of 96% compared to clinical diagnosis (76). Standardized protocols for TAUS delineated by the TABUL study have been widely used in the UK and Europe (40, 46) but not in the United States (58). The main limitation regarding ultrasound is that it is highly operator-dependent: in the TABUL study, for example, the intraclass correlation coefficient for agreement was 0.61 among sonographers, although it should also be noted that the correlation coefficient was 0.62 among pathologists for TAB in that study (see also **Table 2**; 58, 62). Due to its non-invasive nature, ultrasound examinations provide the added benefit of examining the full length of both temporal arteries as well as the axillary arteries, offering greater sensitivity possibly due to longer persistence of abnormalities in larger vessels compared to temporal arteries (62). Furthermore, TAUS has been found to be cost effective relative to TAB (83).

**Table 2:** Diagnostic Testing for GCA.

Diagnostic Method	Sensitivity	Specificity	Intra-observer Correlation	Reference
TAB	39%	100%	0.62	Luqmani et al. (62)
TAB	61%	98%	Not reported	Dua et al. (77)
TAB	69%	100%	Not reported	Hansen et al. (78)
Ultrasound	77%	96%	0.61	Luqmani et al. (62)
Ultrasound	63%	79%	Not reported	Hansen et al. (78)
MRI	78.4%	90.4%	0.676	Klink et al. (79)
MRI with “black-blood” protocol	80%	100%	0.83	Rodriguez-Régent et al. (80)
FDG PET/ CT	71%	91%	0.65	Sammel et al. (81)
FDG PET/ CT	85%	83%	0.84	Grayson et al. (82)

TAB, Temporal artery biopsy; MRI, Magnetic resonance imaging; FDG PET, 18F-Fluorodeoxyglucose positron emission tomography; CT, computed tomography.

Magnetic resonance imaging (MRI) can also be used in the diagnosis of GCA—findings include mural edema in the temporal arteries, as well as the ability to visualize other arteries (84). A meta-analysis of 6 studies estimated sensitivity of 73% and specificity of 88% for MRI compared to clinical diagnosis (76), suggesting that MRI is comparable to TAB in diagnostic value, and may spare TAB in patients with normal MRI findings. Furthermore, MRI is much less operator dependent compared to ultrasound (62). Potential drawbacks of MRI include limited availability and cost, as well as the limited detection of mural inflammation once steroid therapy has started (85), although more targeted protocols may allow for faster scan times and potentially reduced costs. MRA or CTA has also recently been recommended for all new GCA diagnosis given the high rate of aortic involvement (45, 86).

“Black-blood” is an MRI vessel wall imaging technique that creates high-contrast images of blood vessel walls that can be particularly useful for diagnosis of GCA. One study evaluating the diagnostic use of fat-suppressed 3D T1-weighted black-blood MRI within 48 hours of steroid initiation reported clear visualization of arterial walls and identification of mural enhancement in GCA patients (80). Images were evaluated by 2 neuroradiologists and compared to clinical diagnoses of GCA, resulting in a sensitivity of 80%, specificity of 100%, and an inter-observer agreement of 0.83. Another study assessed the diagnostic use of MRI black-blood for detecting posterior ciliary artery (PCA) involvement in patients with GCA and AAION (87). 13 out of 18 GCA patients showed positive findings for AAION with MRI, with MRI black-blood findings displaying contrast enhancement around the optic nerve and adjacent orbital fat along the course of PCAs (87). Bilateral involvement of AAION was apparent in 12 out of 13 MRI black-blood cases, compared to only 6 out of 13 cases identified by ophthalmoscopy (87). These studies suggest that MRI black-blood may supersede ultrasound in its capacity for clearer readings and higher inter-observer agreement, and may be able to detect early ischemia even before profound vision loss occurs. Most centers with 3T MRI machines are able to perform black blood protocols and one should discuss with the radiologist to ensure the proper sequences are obtained.

18F-Fluorodeoxyglucose positron emission tomography (FDG PET)/computed tomography (CT) can be used to detect large-vessel vasculitis in the aorta and the subclavian, carotid, iliac, and femoral arteries, but has generally not been recommended as first-line for the assessment of cranial arteries (88). FDG PET/CT offers advantages in its ability to visualize all inflamed vessels in the body, as long as they are big enough to be detected. Potential drawbacks of using FDG PET/CT include its higher cost, the substantial amount of patient preparation, and exposure to ionizing radiation (88). Spatial resolution has improved with newer “time-of-flight” scanners such that PET/CT can reliably detect changes in cranial vessels (89), and recent studies have shown better results when scans are done within 72 hours of glucocorticoid treatment (81). One recent prospective study using time-of-flight PET/CT within 72 hours of glucocorticoid treatment revealed a sensitivity of 71% and specificity of 91% when using clinical diagnosis as the standard, with interobserver reliability of 0.65 (81).

## Treatment

High-dose glucocorticoids have been the mainstay in treating acute GCA. The 2018 EULAR recommendations suggest 40-60 mg/day prednisone equivalent in treating acute GCA, 15-20 mg/day within 2-3 months, and ≤5 mg/day after 1 year to avoid relapse (67). In emergent situations where GCA patients present with acute visual loss or amaurosis fugax, high dose (up to 1 g/day) intravenous methylprednisolone is recommended for up to 3 days, then transitioning to 1 mg/kg oral prednisone (67). Specialized centers for diagnostic work-up within 24 hours of presenting symptoms of GCA, called “fast-track clinics” in Norway and the UK, have been shown to reduce the risk of permanent visual loss, highlighting the importance of rapid diagnosis and treatment of GCA (90, 91). Overall, there has been a decrease in permanent blindness associated with GCA over the past several decades, likely due to earlier recognition and treatment (48).

Because of the high incidence of visual symptoms, ophthalmologists play a key role in the early/acute treatment of GCA. Rheumatologists generally manage the long-term steroid dosing or steroid-sparing agents. The effectiveness of corticosteroid therapy is often monitored by measuring ESR and CRP over time (44), but these inflammatory markers may not consistently reflect disease activity (75). Patients usually require at least 2 years of corticosteroid therapy, and most are able to taper off by 5 years after diagnosis, but relapse of GCA is common when glucocorticoids are tapered (92). While there is no major consensus on treatment regimens for relapse, the EULAR recommendations suggest treating “major relapse”, defined by clinical symptoms of jaw claudication, visual symptoms, visual loss, scalp necrosis, stroke, limb claudication, or active aortic inflammation, with high dose glucocorticoids of 40-60 mg/day (67). Minor relapses, defined by recurrence of active disease without fulfilling the criteria for a major relapse, are recommended to be treated 5-15 mg above the last effective dose of glucocorticoids.

Long-term treatment with high-dose corticosteroids carries high risk of complications, including but not limited to osteoporosis, diabetes, cardiovascular disease, and glaucoma. In light of these potential complications, the role of glucocorticoid-sparing agents are being studied in the maintenance of GCA remission (51). Methotrexate (MTX) is an antifolate that interferes with DNA synthesis. It is widely available, affordable, and oral, but outcomes associated with MTX in treating GCA have been variable. A meta-analysis of 3 randomized placebo-controlled trials was performed showing that MTX reduced corticosteroid dose by 842 mg within 48 weeks, and had a higher probability of sustaining discontinuation of corticosteroids for ≥24 weeks (93). However, further research is necessary in determining whether methotrexate has a clear benefit in the treatment of GCA.

Tocilizumab (TCZ), a humanized monoclonal antibody that blocks the interleukin-6 receptor, is given by subcutaneous injection every 1-2 weeks, and is the first FDA-approved therapy specific for GCA. Increased interleukin-6 (IL-6) levels are thought to induce acute phase response and systemic manifestations of GCA (94). Randomized controlled trials have shown that tocilizumab has significantly lower rates of relapse and lower exposure to glucocorticoids (60). A randomized placebo-controlled phase 3 clinical study called the Giant-Cell Arteritis Actemra (GiACTA) trial showed that 162 mg of tocilizumab weekly or every other week in combination with a prednisone taper over 26 weeks was superior in sustaining remission, compared to a placebo and prednisone taper over 26 weeks, or a placebo and prednisone taper over 52 weeks (95). Furthermore, adverse events were lower the in tocilizumab group (15% vs 22%) although the study duration was too short to potentially capture long-term side effects of glucocorticoids or tocilizumab (96). A recent paper of real-world use supported the efficacy of tocilizumab in sustaining remission, and specifically noted no new flares associated with vision loss once patients were started on tocilizumab (97).

There are additional targeted agents against IL-6/IL-6R as well as targeted therapies against T-cells which are being studied in GCA (abatacept and ustekinumab), suggesting that the treatment landscape may be quite different in a few years.

## Discussion

Giant cell arteritis is the most common form of vasculitis in adults, with frequent and often devastating ophthalmic manifestations (50). Recent advances in our understanding of GCA diagnosis and management hold the promise of better detection and better treatment outcomes for patients.

Recent epidemiologic studies have demonstrated that incidence rates of GCA in African American populations are higher than previously thought. Variation in the definition of Hispanic underlies the conflicting study results regarding GCA rates in that very heterogeneous population. The largest incidence studies come from European nations with nationalized healthcare systems that facilitate population level analysis. The increasing ability to explore big data in the United States whether through the Intelligent Research in Sight (IRIS) registry or insurance/Medicare databases may help gather more insights on GCA and related ophthalmic complications in more diverse populations.

The ophthalmologist often plays a key role in suspecting the diagnosis of GCA, and that is unlikely to change despite advances in technologies to support that diagnosis.

Once GCA is suspected, a thorough ophthalmic exam and testing including visual fields, OCT, OCT-A, FFA, and/or ICG can help support the diagnosis, and emerging technologies such as laser speckle flowgraphy are being studied. Noninvasive radiologic imaging such as TAUS, MRI, or PET may be used to confirm or exclude the diagnosis, evaluating the entire length of the temporal arteries on both sides, as well as other vessels. Algorithms for stratifying risk/pre-test probability can be applied to improve diagnosis. Advances in MR and PET protocols as well as deep learning algorithms for ultrasound interpretation will likely further improve the sensitivity, specificity, and clinical utility of these modalities. MR or CT imaging is also increasingly used to screen for aortic as well as cerebrovascular complications. TAB nevertheless remains the gold standard for many physicians to confirm a diagnosis of GCA; the majority of recent studies and guidelines suggest a post-fixation TAB length of 1cm is sufficient, especially if the pathologist samples extensively along the entire length of submitted artery.

Once the diagnosis is confirmed, long-term glucocorticoids remain the backbone of GCA therapy for now, but tocilizumab and other agents may be used to reduce overall glucocorticoid exposure and its associated complications, and additional new therapies are being investigated. Further studies on dosing and longterm adverse effects are needed, and advances in immunology may provide more useful biomarkers than ESR and CRP to diagnose and/or follow treatment effect. Collectively, these advances are changing the way we perceive and manage this common but challenging condition.

## Author Contributions

JC conceived the idea and scope of the review. EY and JC both performed literature review and wrote the manuscript. All authors agree to be accountable for the content of the work. All authors contributed to the article and approved the submitted version.

## Conflict of Interest

The authors declare that the research was conducted in the absence of any commercial or financial relationships that could be construed as a potential conflict of interest.

## Publisher’s Note

All claims expressed in this article are solely those of the authors and do not necessarily represent those of their affiliated organizations, or those of the publisher, the editors and the reviewers. Any product that may be evaluated in this article, or claim that may be made by its manufacturer, is not guaranteed or endorsed by the publisher.
